# 

**Published:** 1866-01

**Authors:** 


### INDEX OF HALL'S JOURNAL OF HEALTH.

### VOL. XIII, 1866.

| | PAGE. |
|---|---|
| Animal Food | 159 |
| Accidents | 244 |
| Alexander, James W. | 263 |
| Air, Pure | 273 |
| Airing Chambers | 276 |
| Burns | 88 |
| Breathing, | 133 |
| Biliousness | 139 |
| Bronchitis | 169 |
| Beautiful Sayings | 186 |
| Bed, Going to | 196 |
| Boils | 212 |
| Butter, Rancid | 239, 235 |
| Boarding House Life | |
| Burning Feet | 288 |
| Cholera, Cause of | 1 |
| " Symptoms | 2 |
| " Cure | 5 |
| " Prevented | 123 |
| " Laws | 129 |
| " Fear of | 130 |
| " Certainties | 204, 209 |
| " Treatment | 225 |
| Cellars | 123 |
| Chimneys | 63, 135 |
| Chambers | 80, 276 |
| Coughing | 113 |
| Cleanliness | 19, 136 |
| Cheerfulness | 146 |
| Consumption | 182 |
| Comfort | 191 |
| Cigars Fatal | 213 |
| Clergymen warned | 236, 274 |
| California | 251 |
| Courting | 272 |
| Chilled, Getting | 274 |
| Cold Feet | 287 |
| Cleaning Shoes | 288 |
| Disease and Sight | 142 |
| " Its causes | 184 |
| Disinfectants | 195 |
| Diarrhœa | 196 |
| Dysentery | 229 |
| Emergencies | 131 |
| Eight Hours a Day | 161 |
| Epilepsy | 173 |
| Eating by Rule | 192 |
| Eye Sight | 194 |
| Early Rising | 237 |
| Farmers' Houses | 53 |
| " Where to Build | 53 |
| " Their Plan | 53 |
| Foul Odor | 117 |
| Filth | 136 |
| Fruits, Preserved | 164 |
| Food, Items | 185 |
| Fever and Ague | 200 |
| Fire Places Open | 219 |
| Family Relation | 247 |
| Fireside | 255 |
| Feet in Travelling | 287 |
| " Care of | 288 |
| Games of Skill | 187 |
| Gunpowder marks | 207 |
| Grunting | 277 |
| Great mistake | 277 |
| House Walls | 77 |
| Horses | 93 |
| Hydrophobia, | 235, 108 |
| Hygiene Moral | 145 |
| Hair Specifics | 179 |
| Hair Wash | 243 |
| Ice Houses | 85 |
| Imaginations | 210 |
| Ice Preservers | 241 |
| Immortalities Dawn | 274 |
| Inflammation of Lungs | 273 |
| In-door-Dangers | 275 |
| Jesting | 147 |
| Jacob's Dream | 253 |
| Kitchen | 80 |
| Lard Purified | 235, 239 |
| Lung Fever | 273 |
| Miasm | 239, 55 |
| Measures | 137 |
| Medicinal Terms | 138 |
| Moral Hygene | 145 |
| Mental Developement | 176 |
| Mouth, Cleaned | 207 |
| Madstone | 235 |

| | PAGE. |
|---|---|
| Mosquitoes | 235 |
| Morality | 256 |
| Marriage | 272 |
| New York Health | 111 |
| Night and Disease | 142 |
| Newspaper Receipts | 160 |
| Potatoes | 109 |
| Preaching Easily | 118 |
| Pork Disease | 140 |
| Politeness | 146 |
| Pianos | 221 |
| Pulse | 240 |
| Paine, Thomas | 259 |
| Pickles | 271 |
| Pneumonia | 273 |
| Quarantine | 127 |
| Repentance | 209 |
| Restless, The | 254 |
| Room, Temperature | 276 |
| Shade Trees | 88 |
| Stables | 89 |
| Surprise Parties | 103 |
| Shams | 106 |
| Servants Wanted | 115 |
| Surgery Extempore | 132 |
| Symptoms | 134 |
| Suicide | 180 |
| Sabbath Days | 188 |
| Stupidities | 190 |
| Summer Health | 208 |
| Sun Stroke | 211 |
| Shut the Mouth | 276 |
| Shoes, Waterproof | 284 |
| Shoes, Varnish | 286 |
| Shoes, Cleaned | 288 |
| Thichiniasis | 140 |
| Tomatoes | 240 |
| Teeth | 264 |
| Utilities | 158 |
| Water-closets | 75 |
| Water Conveniences | 73 |
| " Purified | 243 |
| Wells | 79 |
| Water Pipes | 91 |
| Weights | 243, 137 |
| Winter Diseases | 273 |
| Women Overworked | 275 |
| Water-proof Shoes | 286 |

### NOTICES.

To SOUTHERN SUBSCRIBERS in the good old times of light taxes, cheap living and universal and uninterrupted prosperity we give notice, that mail facilities ceased just after the July number of 1861 was distributed ; we kept the subsequent numbers from August to December, both included, bound in one cover ; they will be sent to each subscriber who will send us their present address.

The contents of the Journal of Health from January 1866 to July inclusive are :

What is Cholera?
Its very first Symptoms.
What to do.
Signs of Recovery.
Danger of Stimulants.
Danger of Self medications.
Homœopathic Treatment.
Farmer's Houses.
Where to Build.
Miasma and its Laws.
Cellars in Dwellings.
Smoky Chimneys.
Water conveniences.
Water Closets.
Ice Houses.
Stables.
Kitchens.
Chambers.
Shade Trees.
Barns.
Water Pipes.
Crazy Farmers, Why.
Wives Overworked.
Daughters ill Health.
Surprise Parties.
Shams.
Potatoes as Food.
To stop Coughing.
Foul Odors.
Preaching Easily.
Domestic Cleanliness.
Ventilation.
Laws of Cholera.
Quarantine.
Cholera Prevented.
Fear of Cholera.
Emergencies.
Extemporaneous Surgery.
Curiosities of Breathing.
Symptoms.
Cellars.
Filth and Purity.
Weights and Measures.
Medical Terms.
Biliousness.
Trichiniasa.
Night Work.
Night and Disease.

All new subscribers must begin with the January number. The January and February numbers are taken up with the subject of the Cholera, and will be sent post paid for 30 cents ; the object of the article is to teach the reader to know what are always the first far off symptoms of Cholera ; when he will be in reality cured, without any medicine whatever if these symptoms are *first* attended to ; what are the more advanced symptoms ; and what is considered the most infallible remedy at this state, applicable to all cases, as a means of arresting the disease until a physician can be called ; the folly of *taking* anything as a preventive of Cholera ; the reason why a so-called preventive will certainly increase the chances of an attack; the certain sign of commencing recovery ; the absolute importance of securing the services of a physician in all cases, where attention was not given to the first symptoms ; how easy it is to know these first symptoms ; the importance of remembering that, as the Cholera, if it comes this year, may assume a different phase, from that of former times, it is not safe to rely on any old remedy, nor to rely on any one's advice but that of a

